# Supplementary material for: Humans best judge how much to cooperate when facing hard problems in large groups
Source: Sci Rep. 2019 Apr 2;9:5497. doi: 10.1038/s41598-019-41773-2 (PMC6445098; doi:10.1038/s41598-019-41773-2)
Supplement: Supplementary file 1 — SUPPLEMENTARY INFORMATION [file 41598_2019_41773_MOESM1_ESM.pdf]

HUMANS BEST JUDGE HOW MUCH TO COOPERATE WHEN FACING HARD PROBLEMS IN LARGE GROUPS
SUPPLEMENTARY INFORMATION

Andrea Guazzini, Federica Stefanelli, Enrico Imbimbo, Daniele Vilone, Franco Bagnoli, Zoran Levnajić

February 26, 2019

Each section of this Supplement is devoted to extending and completing a specific point mentioned in the main paper. Sections are organized to follow the exposition in the main paper.

Contents

1 Operationalization of problem complexity in experimental psychology **1**

2 Other technical details about the experiment **2**

3 More on design and implementation of the computer model and simulation **3**

4 Normalization of agent fitness values **3**

5 Comparison of empirical and simulated values of group bonus **4**

6 Analysis of the role of psychological variables **4**

7 Analysis of the role of learning effects **6**

1 Operationalization of problem complexity in experimental psychology

Operationalization of problem complexity is a highly non-trivial challenge in experimental psychology [1, 2, 3, 4], specially when the problem is to be perceived as equally complex by everyone involved. The complexity of the task (problem) is usually defined as the knowledge needed to complete each step necessary to solve it [5]. The more complex is the task, more steps are necessary to solve it, and more knowledge is required to overcome each step. Knowledge is organized as a series of mental schemes [6, 7], where each scheme is a stored concept. So, in order to solve a problem, people have to activate several mental schemes, which are built on the basis of past experience. Since each human has different life experiences, the mental schemes that they build are different for each person. For one person the tasks are more or less complex on the basis of the schemes that he/she has and can use to solve them. So, since an arbitrarily selected human person will have individual and unique schemes, the complexity of the problems put in from of him/her will be perceived as of certain level of complexity, unique for him/her. For this reason the research about decision-making has usually not considered the individual differences in the resolution of the task, but focuses on the influence of decision variables on the decision-maker's behavior [8]. In particular, the two variables most taken into account are the framing of a decision task [9] and the complexity of the decision [10]. Studies that pay attention to interpersonal differences tend to consider their effect on the variance of decision-making as separate from the demands of the task and from its complexity [8]. So, if we wish to separate individual and task effects, then we

should logically expect to describe tasks independently of individuals who perform the task. Since this equal perception is what we chiefly aim at in this paper with our experimental setting, the price we pay is that our participants are not solving real problems. Instead, they are playing a probabilistic game, where "probability to solve a problem" is a proxy for problem complexity. Furthermore, realizing actual scale of problem complexity is even harder within an already simplified model of a given social phenomenon or interaction among humans. It is important to stress that here we are interested in modeling the *effects* that problem complexity has on the behavior and abilities of the participants, and not in modeling the problem complexity *per se*. The difficulty and the complexity of a problem are, from a theoretical point of view, rather different attributes, because the former relates to the subjective perception of the problem, while the latter concerns – at least in principle – an objective property [11]. In any case, in our experiment, we believe we can realistically work under assumption that humans have a lower probability to solve a more complex problem than a less complex one, which in fact has been shown in the previous studies [1]. For this reason, we set our model directly in the format in which a more complex problem is modeled as a low probability of receiving the reward, whereas a less complex problem is represented as a higher probability of getting the same reward. In fact, from economic point of view this formulation represents the same dilemma as if the problem was real: how much resources should I invest in a problem that is (or appears to be) *that hard*? Am I better off cooperating with other players or not? Most importantly, this approach allows for our experiment to be doable in present way, so that the discrepancy between optimal and real behavior can be consistently measured and interpreted.

2 Other technical details about the experiment

The software for our experiment (game) was developed using *Google Apps Script* based on the programming language *JavaScript*. It involved both the on-line interface for playing and the underlying database where the results (decisions) were stored. The experiment took place in a computer lab, with computers suitably arranged. Privacy of each participant in making his/her decisions was assured and nobody was able to see anyone's else computer screen. The participants were not allowed to use smartphones and other communication devices during the experiment. The experimenters made sure that participants clearly understood both the technical rules of how the game is played, as well as the goals they should strive to reach in playing the game. The surveys with psychological measures were completed before the beginning of the game. Once the experiment concluded, stored data was pre-processed to check the consistency of statistical properties before the actual data analysis. The reason we chose exactly 11 rounds to make up one game is because we estimated that this number will offer the best balance between the possible intuitive tendencies of players to cooperate and to compete. This then makes both tendencies equally present (on average), so our results are not bias on this. Much smaller number of rounds would render the group bonus GB meaningless, since not enough accumulation would be be generated. Much larger number of rounds could make the players "bored" of playing on the same S and R, which would again make the results bias. In figures that follow we show the screen that players were looking at while playing. It was made to allow for simple keeping track of one's score and decision making.

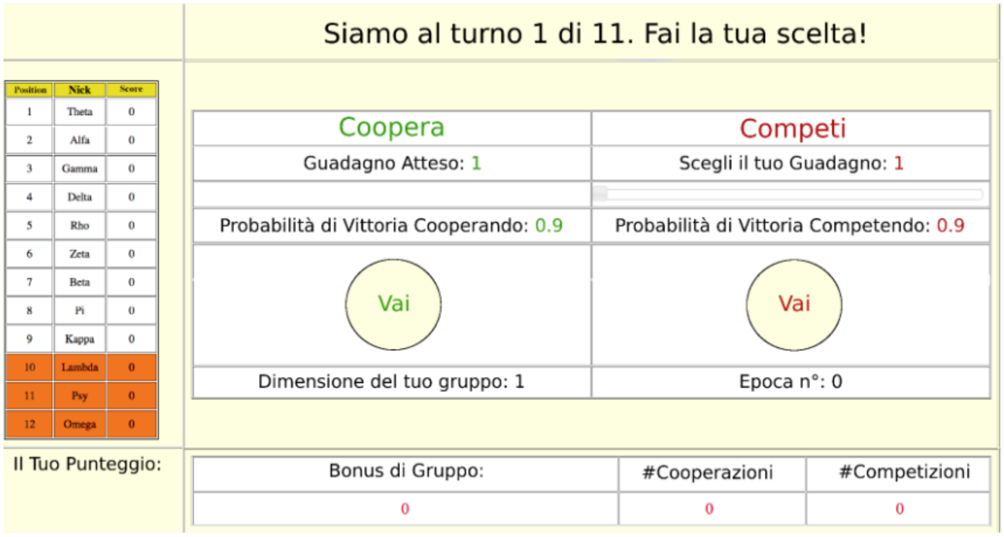

Supplementary figure S 1: **The original player interface of the software used in the experiment. Since all players declared themselves to be native Italian speakers, we used only Italian language in this interface for easier playing. Next figure shows the English translation of this interface.**

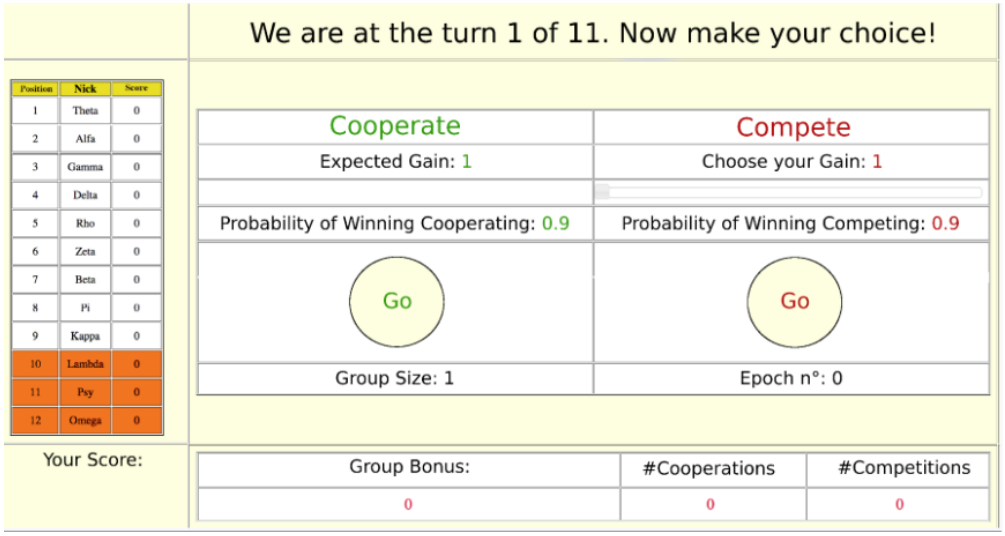

Supplementary figure S 2: **The English translation of the player interface used in the experiment.**

3 More on design and implementation of the computer model and simulation

To quantify the usefulness of social heuristics, we need to compare the human behavior observed in the experiment with the theoretically optimal behavior under the same conditions. However, reproducing the "same" conditions as in a real-world experiment is difficult. Still, our entire experiment was designed so that it can be precisely translated into a computer simulation with evolving agents modeling human players. Letting the agents evolve in a competitive environment (similarly to a genetic algorithm), we were able to exactly compute the best possible payoff associated with the optimal strategy [12]. We however did this in approximation that an evolving agent has a fixed collaboration probability. Specifically, we used an evolutionary algorithm (similar to a genetic algorithm) to compute the extreme behavior (strategy) that maximizes the agent fitness AF (we call our agents 'evolving agents' for this reason). This was implemented using a large population of evolving agents that played the same set of tasks (problems) as real players for the same set of difficulty levels R and group division schemes S. Each agent i is defined by a parameter q_i which models the cooperation probability. After each game the algorithm eliminates the 20% of agents with the worst AF, replacing them with new agents with random values of q. Repeating this process many times (until a stable equilibrium was reached) we extracted the best level of cooperation q that maximizes AF, for all R and S. We also numerically confirmed that no other homogeneous values of q leads on average to higher AF. This result allows the comparison between the actual average human cooperation C and the "best" level of cooperation (average of q, called C_{best}), which leads to the highest attainable AF (called AF_{best}). The algorithm implemented in this study is practically identical to the one published in [12], where we refer the reader for a more complete description.

4 Normalization of agent fitness values

Theoretical and real values of AF cannot be immediately compared, since due to the evolutionary nature of simulations the two scoring schemes are not normalized in the same way. In order to make an interpretable comparison of two values of AF, we conducted two additional computer simulations reproducing the same experimental environment. In the first new simulation we fixed the cooperation level to C_{best}, the theoretically best collaboration level coming from the original simulation. In the second new simulation we fixed the collaboration level to the average value of C obtained experimentally for human players. We ran these two simulations without evolutionary optimization, aiming them exclusively to compute the final values of AF in two cases. The result of the first new simulation we define as AF_{best} – the maximal attainable agent fitness corresponding to the scenario of an agent constantly playing at the optimal collaboration level C_{best}. The result of the second new simulation we define as the average AF obtained when a player constantly plays at the average empirically observed human collaboration level C. This pair of new simulations was run for each combination of values of R and S, yielding AF_{best} and AF for each R and S. The difference between thus obtained AF_{best} and AF is interpretable as unbiased comparison between the cumulative payoffs of human players and evolving agents, which we analyze in the main text. Note however that these new simulations are done in imperfect approximation that all players/agents always cooperate with the same fixed probability, fixed either to C_{best} or C. The fact that humans somewhere earn slightly more than the theoretical model comes from the imperfection of this

approximation. See also Discussion in the main text, where we examine when and how it is adequate to approximate a player's behavior with as a fixed cooperation probability.

5 Comparison of empirical and simulated values of group bonus

In order to compare the group bonuses for humans and evolving agents we repeat on the simulations previously done for the purpose of comparing agent fitness, which are described in the section above. That is to say, we repeat the same simulation as described in the section above, but this time instead of AF_{best} and AF we compute the values of GB_{best} and GB, by fixing the collaboration levels to C_{best} and C, respectively. This is again done separately for each combination of R and S, leading to interpretable and unbiased comparison between maximal attainable GB_{best} and actual human GB. Recall that the original simulation leading to C_{best} was done for maximization of AF (and not GB), so here we examine how good GB such optimization leads to. The results are shown in Fig.3 using the same scheme as in the Fig.1 in the main text. Similarly to the case of AF examined in the main text, we find a clear correlation between GB and both R and S. Specifically, we find a pattern very similar to what found for AF: when dealing with simple problems and when playing in small groups, humans generate considerably smaller GB than they could. As problems get harder and/or as groups get larger, the group bonus generated is gradually closer to the maximal values. When confronting hardest problems or when playing all together in a single group, humans generate the maximal attainable GB.

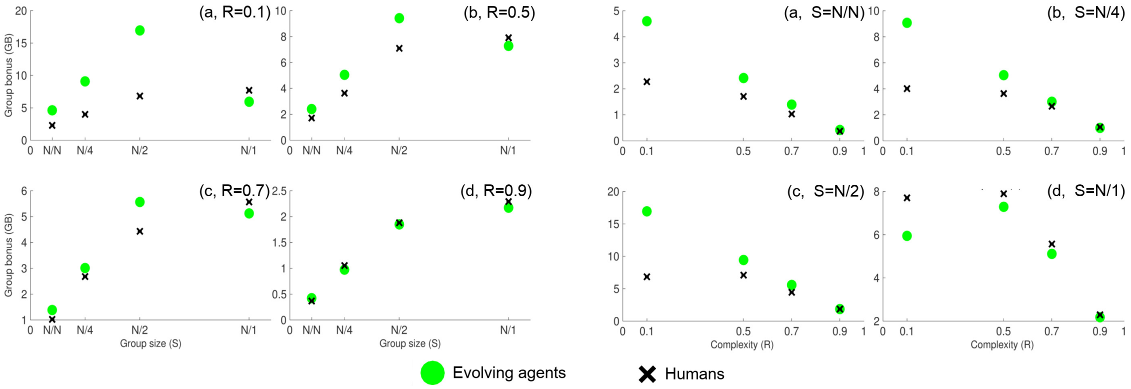

Supplementary figure S 3: **Comparison of the experimental and the simulated values of group bonus.** Comparison of experimental GB (black crosses) and best attainable GB_{best} (green circles), obtained as explained in the text. Left panel: four plots (a)-(d) show the comparison over four values of problem complexity R. Within each plot we show the values for four group sizes S, where N/N indicates the group composed of a single player, while $N/4$, $N/2$ and $N/1$ respectively indicate the divisions into 4, 2 and 1 group, as done in the Fig.1 in the main text. Right panel: four plots (a)-(d) show the same comparison, but this time over four group sizes S. Results we find are similar to those for agent fitness: when dealing with simple problems and when playing in small groups, humans generate much smaller GB than they could. This improves as problems get harder and/or as groups get larger. Finally, when facing hardest problems or when playing all together in one group, humans generate the maximal attainable GB. The fact that humans somewhere appear to earn slightly more than theoretical model is an artifact due to statistical nature of the corresponding simulations. All differences are statistically significant.

6 Analysis of the role of psychological variables

In order to evaluate the possible role of individual psychological variables on cooperative behavior of players, each participant was asked to complete a survey composed of two parts. The first part consisted of usual socio-demographical questions: gender, age, number and members of the household, education level, civil status, number and kind of community membership and social networks used. The second part involved five standard psychological questionnaires:

- *The Five Factor Adjective Short Test* (5-FasT), a personality inventory test composed by five sub-scales: Neuroticism, Surgency, Agreebleness, Closeness and Conscientiousness. The inventory was composed of a total of 26 items on a five-point scale (1 = strongly disagree, 5= strongly agree) [13],

- *Honesty-Humility* sub-scale, part of the *HEXACO* personality inventory. The sub-scale was composed of a total of 16 items on a five-point scale (1 = strongly disagree, 5= strongly agree), four for each of the following dimensions: sincerity, fairness, greed-avoidance and modesty, [14];
- *State* sub-scale from the *State-Trait Anxiety Inventory* (STAI Form Y-1) used in order to evaluate the anxiety state of a participant at the moment of the experiment. The sub-scale was composed of a total of 10 items on a four-point scale (1 = not at all, 4= very much so) [15],
- *General Self-Efficacy Scale* that is correlated to emotion, optimism and work satisfaction. Negative coefficients were found for depression, stress, health complaints, burnout, and anxiety. The scale was composed of a total of 10 items on a four-point scale (1 = not at all true, 4= exactly true) [16],
- *Social Community* scale, part of the *Classroom and School Community Inventory*, a measure suitable to evaluate the sense of community of participants. The scale was composed of a total of 10 items on a five-point scale (1 = strongly agree, 5=strongly disagree) [17].

We implemented the calculation of the standard linear regression using as the dependent variable the average probability of cooperation C (for each combination of R and S), and as the independent variables the complexity of the problem R and the survey-measured psychological variables for each player. We found the linear regression to predict the tendency to collaborate from the psychological variables as $F(2, 214) = 65.167, p < 0.01$, with $R^2 = 0.233$ (see Table 1).

Supplementary Table S 1: Best linear regression model for cooperation probability C

R	Adj-R^2	F
.233	.233	65.167^{**}

$^{**}p < .0001$

Here F stands for the statistical value of the usual F-test. In the linear regression models the F-test compares the fits of different linear models. According to the best linear model, the best predictors of the average probability of cooperation C for an average player were as follows, in descending order:

- the size of the group S, explaining 35% of the variance,
- the complexity of the problem R, explaining 32% of the variance,
- the individual honesty, explaining about 6% of the variance, measured by the *Honesty-Humility* sub-scale, part of the *HEXACO*,
- some of participant specific personality characteristics, such as the degree of neuroticism, explaining about 6% of the variance, measured by the *The Five Factor Adjective Short Test* (5-FasT),
- anxiety that players perceive in the exact moment when doing the task (problem), explaining about 5% of the variance, measured by the *State* sub-scale, of the *STAI Form Y-1*,
- individual's attachment to the community, explaining about 5% of the variance, measured by the *Social Community* scale, part of the *Classroom and School Community Inventory*,
- the dynamism and conscientiousness, explaining about 5% of the variance, measured by the *The Five Factor Adjective Short Test* (5-FasT),

with other less relevant factors cumulatively accounting for the remaining percentages. See Table 2 for more details on these coefficients.

Supplementary Table S 2: Predictors coefficients of the best model for Cooperation probability

Predictor	Stand. Coefficient	t	Significance
Group Size	$\beta_7 = -.346$	-16.363	$p < .0001$
Complexity of the task	$\beta_8 = .316$	14.931	$p < .0001$
Hexaco Honesty	$\beta_4 = .068$	2.870	$p < .001$
Neuroticism	$\beta_1 = .056$	2.200	$p < .05$
STAI	$\beta_5 = -.051$	-1.992	$p < .05$
Surgency	$\beta_2 = -.056$	-2.507	$p < .01$
Conscientiousness	$\beta_3 = .048$	2.226	$p < .05$
Sense of Community	$\beta_6 = .049$	2.277	$p < .05$

As clear from the table, variables R and S are indeed the key to this problem, since they jointly account for 67% of the variance. We also look examined these results in the light of the SIDE Model, which focuses on the cognitive processes by which CMC (computer-mediated-communication) communicators use social identity variables and social category information to make inferences and take decisions. According to SIDE this should result in subject de-individualization, which leads the agents to behave in accordance with the local norms and perceived characteristics of the environment, rather than in accordance whit their personal characteristics [18]. In this light, human players in our experiment show no considerable effect of the psychological and demographic variables on the collaborative decisions. In particular, this has been verified in the absence of significant differences due to gender as a possible additional predictor variable of the tendency for cooperation. This result is in agreement with the most recent literature on the subject and shows how the two variables R and S indeed explain the largest part of this variance.

7 Analysis of the role of learning effects

In order to determine whether learning over time had an effect in the participants' tendency to cooperate, in addition to group size S and problem complexity R we introduce another factor called $Time$ as follows. Each participant was asked to play 8 games, each game composed of 11 decisions (rounds). Those 8 games were always composed of two series of 4 games, each series with a fixed problem complexity R. The group size S was always changed along the four different sizes (see main text). In order to identify and evaluate possible learning effects in our experiment, we now introduce the observable $Time$, which indicates for each participant the sequence of time intervals needed for that participant to make his/her cooperative decisions. We hence obtain for each participant $11 \times 8 = 88$ different records for the $Time$ variable. Basically, we consider the time it took for each among 216 players to make each cooperative decision, for each combination of R and S. In this way we can examine if and how the cooperative behavior changes as a function of the elapsed time before taking the decision to cooperate or not to cooperate. Therefore, based on this we can either verify or exclude the presence of any systematic rational reasoning that can be attributed to players learning during the course of experiment how to play in their best interest (either individual best interest of group best interest). To this end we use the statistical technique called *Generalized Linear Mixed Models* (GLMM), which is an extension of the standard generalized linear modeling (GLM), in which the linear predictor contains random effects, in addition to the usual fixed effects [19, 20]. GLMM also inherits from GLM the idea of extending linear mixed models to non-normal data. Fitting GLMM via maximum likelihood involves integrating over the random effects. In general, those integrals cannot be expressed in analytical form. The peculiarities of GLMM concerns the possibility to model the fixed and random effects of a pool of continuous and discrete variables, in order to predict the target variables (in our case the discrete variable that regards the event of decision to cooperate or not to cooperate). This method allows also to test the single effects of each variable within the model, as well as their combined effect. In this way, by the virtue of introducing the time dimension among the model variables, allowed us to test possible learning effects as either independent from other variables or combined with them. Applying GLMM, we find the best significant model and present it in Table 3. It confirms that if the three factors - group size S, problem complexity R, and $Time$ - are considered together in the analysis, the variable $Time$ (i.e., the learning effect) has no measurable influence on the probability of cooperation C when compared with the influence of R and S.

Supplementary Table S 3: The parameters of the best model obtained via Generalized Linear Mixed Model procedure (with repeated measures). The significant parameters have degree of freedom (Df) = 1,18806 ($^{***} = p < 0.001$, $^{**} = p < 0.01$, $^{*} = p < 0.05$).

**GLMM best model**			
**Model Precision**	$Akaike^{*}$	**F**	**Df-1(2)**
67.9%	3605.50	120.22^{***}	4(18806)
**Parameter**	**Fixed effect (F)**	**Coefficient**	**Student *t***
Group size S	34.91^{***}	-0.110	-5.91^{***}
Problem complexity R	54.76^{***}	0.161	7.40^{***}
$Time$	1.55	-0.007	-1.24

That is to say, we do not find that learning plays almost any effect in our experiment, meaning that on average, participants in each round make their decisions as if it was the first round for them. As a consequence, we find no significant learning effects on cooperation probability C, neither alone nor in the interaction with others independent variables, which implies that no learning effects influence our players behavior whatsoever. Moreover, in this way we also exclude in the first approximation any systematic effect of the interaction between the experimental factors on the variance taken into account by the GLMM. Therefore, we believe that based on the above analysis we can safely ignore the learning effects in our experiment, as argued in the main text (see Discussion section). This further confirms our

claim that players mostly rely on intuitive (heuristic) processing of information when making cooperative decision in our experiment.

References

[1] Donald J Campbell. Task complexity: A review and analysis. *Academy of management review*, 13(1):40–52, 1988.

[2] Douglas C Maynard and Milton D Hakel. Effects of objective and subjective task complexity on performance. *Human Performance*, 10(4):303–330, 1997.

[3] Celia Moore and Ann E Tenbrunsel. "just think about it"? cognitive complexity and moral choice. *Organizational Behavior and Human Decision Processes*, 123(2):138–149, 2014.

[4] Valerio Capraro and Giorgia Cococcioni. Rethinking spontaneous giving: Extreme time pressure and ego-depletion favor self-regarding reactions. *Scientific reports*, 6:27219, 2016.

[5] Robert E Wood. Task complexity: Definition of the construct. *Organizational behavior and human decision processes*, 37(1):60–82, 1986.

[6] Frederic C Bartlett. *Remembering: An experimental and social study*. Cambridge, UK:Cambridge University, 1932.

[7] Dorothy Tse, Rosamund F Langston, Masaki Kakeyama, Ingrid Bethus, Patrick A Spooner, Emma R Wood, Menno P Witter, and Richard GM Morris. Schemas and memory consolidation. *Science*, 316(5821):76–82, 2007.

[8] Shoshana Shiloh, Shelly Koren, and Dan Zakay. Individual differences in compensatory decision-making style and need for closure as correlates of subjective decision complexity and difficulty. *Personality and individual differences*, 30(4):699–710, 2001.

[9] Amos Tversky and Daniel Kahneman. The framing of decisions and the psychology of choice. *science*, 211(4481):453–458, 1981.

[10] John W Payne. Task complexity and contingent processing in decision making: An information search and protocol analysis. *Organizational behavior and human performance*, 16(2):366–387, 1976.

[11] Peter Robinson. Task complexity, task difficulty, and task production: Exploring interactions in a componential framework. *Applied linguistics*, 22(1):27–57, 2001.

[12] A Guazzini, D Vilone, C Donati, A Nardi, and Z Levnajić. Modeling crowdsourcing as collective problem solving. *Scientific reports*, 5:16557–16557, 2014.

[13] Marco Giannini, Linda Pannocchia, Rosapia Lauro Grotto, and Alessio Gori. A measure for counseling: The five-factor adjective short test (5-fast). *Giornale Italiano di Ricerca e Applicazioni*, 3:384, 2012.

[14] Kibeom Lee and Michael C Ashton. Psychometric properties of the hexaco personality inventory. *Multivariate Behavioral Research*, 39(2):329–358, 2004.

[15] Charles D Spielberger. *Manual for the State-Trait Anxiety Inventory STAI (form Y)("self-evaluation questionnaire")*. Palo Alto, CA:Consulting Psychologists Press, Inc., 1983.

[16] Lucio Sibilia, Ralf Schwarzer, and Matthias Jerusalem. Italian adaptation of the general self-efficacy scale, 1995. Resource document. Ralf Schwarzer web site. Accessed 07 July 2018 from http://www.ralfschwarzer. de/.

[17] Miretta Prezza, Maria Giuseppina Pacilli, Claudio Barbaranelli, and Emanuela Zampatti. The mtsocs: A multidimensional sense of community scale for local communities. *Journal of Community Psychology*, 37(3):305–326, 2009.

[18] Russell Spears, Tom Postmes, Martin Lea, and Susan E Watt. A side view of social influence. In Kipling D. Williams and Joseph P. Forgas., editors, *Social Influence: Direct and Indirect Processes.*, pages 331–350. Philadelphia, PA:Psychology Press, 2001.

[19] Norman E Breslow and David G Clayton. Approximate inference in generalized linear mixed models. *Journal of the American statistical Association*, 88(421):9–25, 1993.

[20] Madsen Henrik and Thyregod Poul. *Introduction to general and generalized linear models*. Boca Raton, FL:Chapman & Hall CRC Press,, 2011.
